# Data mining of iron(II) and iron(III) bond-valence parameters, and their relevance for macromolecular crystallography

**DOI:** 10.1107/S2059798317000584

**Published:** 2017-03-31

**Authors:** Heping Zheng, Karol M. Langner, Gregory P. Shields, Jing Hou, Marcin Kowiel, Frank H. Allen, Garib Murshudov, Wladek Minor

**Affiliations:** a Department of Molecular Physiology and Biological Physics, University of Virginia, Charlottesville, VA 22901, USA; b Cambridge Crystallographic Data Centre, 12 Union Road, Cambridge CB2 1EZ, England; c Center for Biocrystallographic Research, Institute of Bioorganic Chemistry, Polish Academy of Sciences, 61-704 Poznan, Poland; d MRC Laboratory of Molecular Biology, Cambridge CB2 0QH, England

**Keywords:** bond-valence model, metal–organics, oxidation state, Cambridge Structural Database, nonlinear conjugate gradients

## Abstract

The bond-valence model is a reliable way to validate assumed oxidation states based on structural data. It has successfully been employed for analyzing metal-binding sites in macromolecule structures. However, inconsistent results for heme-based structures suggest that some widely used bond-valence *R*_0_ parameters may need to be adjusted in certain cases. Given the large number of experimental crystal structures gathered since these initial parameters were determined and the similarity of binding sites in organic compounds and macromolecules, the Cambridge Structural Database (CSD) is a valuable resource for refining metal–organic bond-valence parameters. *R*_0_ bond-valence parameters for iron(II), iron(III) and other metals have been optimized based on an automated processing of all CSD crystal structures. Almost all *R*_0_ bond-valence parameters were reproduced, except for iron–nitrogen bonds, for which distinct *R*_0_ parameters were defined for two observed subpopulations, corresponding to low-spin and high-spin states, of iron in both oxidation states. The significance of this data-driven method for parameter discovery, and how the spin state affects the interpretation of heme-containing proteins and iron-binding sites in macromolecular structures, are discussed.

## Abstract 2

Using all available metal-containing organic compound structures in the Cambridge Structural Database, a novel data-driven method to derive bond-valence *R*_0_ parameters was developed. While confirming almost all reference literature values, two distinct populations of Fe^II^—N and Fe^III^—N bonds are observed, which are interpreted as low-spin and high-spin states of the coordinating iron. Based on the *R*_0_ parameters derived here, guidelines for the modeling of iron–ligand distances in macromolecular structures are suggested.

## 1. Introduction   

The bond-valence model relates the oxidation number of an atom to its immediate surroundings, and as such has been indispensable in a multitude of structural applications (Brown, 2009 ▸), including the analysis of metal-binding sites in proteins (Müller *et al.*, 2003 ▸). During the investigation of metal ion-binding architectures in proteins (Zheng *et al.*, 2008 ▸, 2014 ▸), we successfully employed the bond-valence model to check the quality of metal-binding site modeling in low-resolution structures. Initially, we used reference literature values for bond-valence (*R*_0_) parameters, which were derived two decades ago from manually curated structures and extrapolated linear relationships between bond-valence contributions (Brese & O’Keeffe, 1991 ▸; Brown & Altermatt, 1985 ▸). However, in cases involving iron and nitrogen, such as structures containing heme, we consistently obtained bond-valence sums that were significantly different from known oxidation states, prompting us to attempt a re-evaluation of bond-valence *R*_0_ parameters for iron-binding sites.

Following the initial publication of bond-valence parameters based on inorganic crystal structures (Brese & O’Keeffe, 1991 ▸; Brown & Altermatt, 1985 ▸), numerous studies have discussed the bond-valence model in the context of small-molecule crystal structures in the Cambridge Structural Database (CSD; Allen, 2002 ▸; Groom *et al.*, 2016 ▸) as well as in the Inorganic Crystal Structure Database (ICSD; Bergerhoff & Brandenburg, 2004 ▸). Some notable results based on CSD data include bond-valence parameters for copper (Shields *et al.*, 2000 ▸), lanthanides (Trzesowska *et al.*, 2004 ▸, 2006 ▸), cadmium (Palenik, 2006 ▸), antimony (Palenik *et al.*, 2005 ▸) and ammonium (García-Rodríguez *et al.*, 2000 ▸). Several more metals were discussed later, as summarized and reviewed by Brown (2009 ▸).

Commonly used macromolecular structure-refinement programs such as *REFMAC* (Murshudov *et al.*, 2011 ▸) and *phenix.refine* (Afonine *et al.*, 2012 ▸) did not use the bond-valence model as restraints. Moreover, the set of metal–ligand bond-length restraints used in these programs were derived from the ICSD, because it contains a more diverse and comprehensive set of metal–ligand interactions. However, proteins and other large biological molecules possess metal-binding environments that are more similar to metal–organics than to inorganic minerals, and so CSD-derived data should be preferable over ICSD-derived data for modeling macromolecular metal-binding sites. To this end, we have chosen the CSD as the starting point for our re-evaluation of bond-valence *R*_0_ parameters, since it contains a vast and diverse set of iron-binding sites in organic crystal structures with a much higher reliability than those observed in macromolecular crystal structures.

Throughout this report, we use the term ‘iron–organic’ to refer to a subset of ‘metal–organic’ compounds, which in turn denotes specifically metal-binding site moieties exclusively containing interactions between metal ions and non-C atoms in organic structures. This is in contrast to ‘organometallic’ sites, which contain bonds between metals and C atoms. Organometallic bonds are rarely observed in biological macromolecules in nature, although there are a few important examples such as cyanocobalamin (Masuda *et al.*, 2000 ▸) and enzymes catalyzing reactions with carbon monoxide (Carlsson *et al.*, 2005 ▸). The atomic ligands analyzed in our work are strongly electronegative (N, O, F, S, Cl and Br) and were chosen partly owing to their biological relevance.

In this spirit, we have built a data-driven procedure to refine the bond-valence *R*_0_ parameters for iron bound to a number of ligand atoms with strong electronegativity (N, O, F, S, Cl and Br). After querying and filtering crystal structures from a recent edition of the CSD (v.5.36, released November 2014), we optimized all iron–ligand *R*_0_ parameters together, which allowed us to include heteroleptic binding sites. Parameters for nearly all bond types agreed with reference literature values, with notable differences for the iron–nitrogen bond parameters in both the iron(II) and iron(III) oxidation states. Furthermore, we find two populations of Fe^II^—N bonds and two populations of Fe^III^—N bonds that are visibly well separated in the distribution of bond-valence sums. It has been noted before (Brown, 2009 ▸) that the bond-valence parameter *R*_0_ derived from different set of references structures differs by as much as 0.05 Å. With this in mind, we cautiously evaluated differences compared with reference values (Brown & Altermatt, 1985 ▸; Brese & O’Keeffe, 1991 ▸), and considered them to be significant only when they were larger than ±0.1 Å. As a control, the same protocol was applied to several other metals (Na, Mg, K, Ca and Zn), and in all these cases our values derived from the same data-driven procedure agreed with the previous parameters within statistical error.

## 2. Experimental   

### 2.1. The bond-valence model   

The bond-valence model relies on the notion that the length of a bond between atoms depends on its valence, *i.e.* the number of electron pairs forming the bond or the electrostatic flux between participating atoms. Another guiding idea, which follows from the above definition, is the bond-valence sum (BVS) rule, which states that the valences of the bonds of an atom should sum up to its oxidation state (which we denote *S*). The sum rule will generally hold in the condensed phase, when there is no charge transfer or strain in the electronic structure around the atom. One may express mathematically and utilize the bond length–valence correlation by adopting various expressions and testing them empirically. The most popular among these is *v_ij_* = exp[(*R*_0_ − *d*_*ij*_)/*b*], where *v_ij_* is the bond valence and *d_ij_* is the length of the bond formed between atoms *i* and *j* (Brown, 2009 ▸). The bond-valence *R*_0_ and *b* parameters are typically obtained by fitting this expression to a set of trusted structures, and will vary with the element types and oxidation states of atoms *i* and *j*. The exponential form cited above has the convenient property that the optimal bond-valence parameter *b* is relatively insensitive to the atom type and binding partners (Brown & Altermatt, 1985 ▸). In most circumstances, a value of *b* = 0.37 Å provides satisfactory results and we assume this value throughout, although the need to adjust *b* in some circumstances has been acknowledged (Brown, 2009 ▸).

Using the standard exponential form, *v*_*ij*_ = exp[(*R*_0_ − *d*_*ij*_)/*b*], for the bond length–valence relationship, the bond-valence sum rule becomes
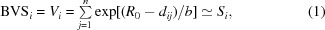
where *V_i_* is the calculated bond-valence sum and *n* is the coordination number of binding site *i*. For homoleptic sites, where all ligand atoms bound to the central atom consist of the same chemical element, *R*_0_ is constant. By setting the calculated BVS equal to the expected oxidation state, *V_i_* = *S_i_*, and treating *b* as a constant, the sum rule can be reorganized into an equation that yields a unique *R*_0_ parameter for any homoleptic binding site *i*,
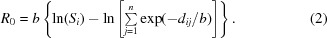



The most common approach to determine the reference *R*_0_ parameters in the literature has been to apply (2) to a number of carefully chosen homoleptic complexes, and then determine the mean and standard deviation of *R*_0_ for each type of metal–ligand pair. This excludes heteroleptic sites, as they involve multiple distinct *R*_0_ values, one for each unique metal–ligand pair (a ligand is an atom type bonded to the central metal). This limitation to homoleptic sites can be addressed in several ways, and the one that we employ in this study (§[Sec sec2.4]2.4) is to fit multiple parameters for a number of sites simultaneously.

### 2.2. Retrieving validated binding sites from the CSD   

For all analyses we used CSD v.5.36 (November 2014; Allen, 2002 ▸; Groom *et al.*, 2016 ▸) as the data source, and *ConQuest* v.1.15 was used for querying and retrieving data (Bruno *et al.*, 2002 ▸). Apart from the normal pre-publication validation and refereeing processes, all data entering the CSD were carefully evaluated for chemical sense and for the internal consistency of coordinates, geometry, unit-cell parameters and space group. This evaluation also includes a probabilistic assignment of atom and bond types (Bruno *et al.*, 2011 ▸). Any residual issues raised by these processes were resolved as far as possible, often in conjunction with the original authors. In order to further minimize the potential for flaws, we started our filtering pipeline by excluding all disordered structures (those with multiple conformations), structures with *R* values above 7.5% and iron sites that contained any iron–carbon bonds.

Queries were constructed individually for each atom without specifying the oxidation state. Iron sites with no bonded atoms (as defined in *ConQuest*/CSD) were also disregarded from further analysis. All atoms within a 4 Å radius of the iron ion were processed (but not necessarily used in the final analysis), including symmetry-related contacts. *ConQuest* also reports symmetry-equivalent iron sites, but these repeats were not used as input. The resulting structures were filtered further using the following criteria.

(i) Ligand atoms contributing less than 5% to the total bond-valence sum were presumed to be outside the first coordination sphere and were discarded. For this, preliminary valence contributions were evaluated using literature *R*_0_ parameters (Brese & O’Keeffe, 1991 ▸). Using this criterion, (i) can be transformed into a distance cutoff *d*_cutoff_ = *R*_0_ − *b*[ln(0.05) + ln(*S_i_*)]. For a ligand of a divalent cation, for example, this is approximately equivalent to a calculated bond valence (*v_ij_*) less than 0.1 and therefore to a distance above *R*_0_ + 0.85 Å.
(ii) Binding sites with ligand atoms other than N, O, F, S, Cl or Br in the first coordination sphere as defined above were discarded, including those bound with carbon ligands.
(iii) Binding sites with only one ligand in the first coordination sphere were removed.
(iv) Any binding site for which the bond-valence sum *V_i_* fell outside of the interval (0, 2*S_i_*), either before or during the optimization described below (using previously published *R*_0_ values or current values during optimization), was automatically removed. The rationale for this is that such extreme bond-valence sums are strongly indicative of an error in the structure or extreme unsuitability for parameterization for chemical reasons.

The set of 79 399 binding sites in 39 706 entries obtained from such a filtering procedure was used to derive *R*_0_ parameters for iron and the other metals used for validation. Although the resulting structures are not guaranteed to accurately obey the bond-valence sum rule, they are much less likely to be outliers. Further improvement of the filtering procedure could involve rejecting binding sites that are not suitable for parameterizing the bond-valence model, for example owing to charge transfer or electron delocalization. In the current study, the assumption is that such sites will contribute to the uncertainty and possible bias of our results and any remaining extreme examples will become outliers. The histogram of bond-valence sums should therefore approximately follow a normal distribution.

### 2.3. Assigning iron oxidation state   

All iron-binding sites that passed the filtering steps (i)–(iii) outlined in §[Sec sec2.2]2.2 were used as input for oxidation-state assignment and subsequent *R*_0_ parameter derivation. The most prevalent oxidation states for iron are ferrous [iron(II)] and ferric [iron(III)]. However, the oxidation states of metal atoms are not assigned within the CSD, although such data do occur in text form in the compound names included in CSD entries. Therefore, the following three-part strategy was adopted to assign iron(II) and iron(III) states to all sites we considered in our pipeline.

(i) If the oxidation state specified in the compound name is clear and unambiguous, then this value was used.
(ii) For other situations, the ligand-template method was used (Shields *et al.*, 2000 ▸). Ligand templates define 250 general donor groups that represent the majority of ligand types commonly observed in metal coordination chemistry. Bond-valence sums were not used in the assignment of iron oxidation states to prevent circular logic.
(iii) For mixed-metal compounds, oxidation-state statistics were used as described and previously implemented (Bruno *et al.*, 2011 ▸).

In around 10% of the cases, this three-part strategy cannot determine the oxidation states and therefore these cases were not considered for further analysis. Besides the expected oxidation states for both ferrous [iron(II)] and ferric [iron(III)] ions, this three-part strategy sometimes resulted in non-integral oxidation states, which were discarded from further analysis. Given the complexity of some of the structures in the CSD, there may still be sites with a misassigned oxidation state; however, the overall empirical error rate in the whole process is likely to be below 3% (Bruno *et al.*, 2011 ▸).

**Figure 1:**
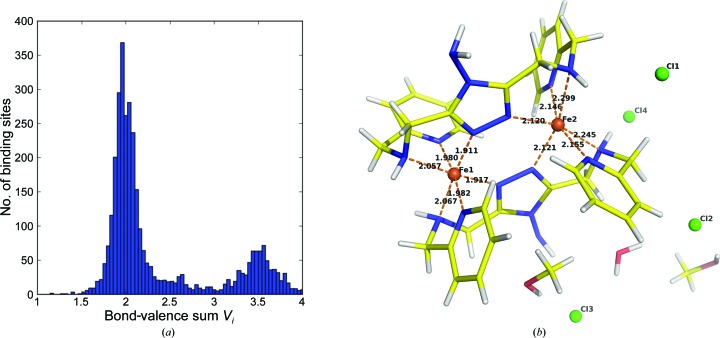
(*a*) Bimodal BVS distribution for iron(II) sites in the CSD using literature *R*_0_ values, with oxidation state assigned by the ligand-template method. (*b*) An example of a crystal structure with two iron(II) sites in different spin states (CSD Refcode ABEQUV). Although both Fe atoms were assigned an oxidation state of 2 and have a coordination number of 6, Fe1^II^—N distances span 1.911–2.067 Å, while Fe2^II^—N distances span 2.146–2.299 Å.

This systematic oxidation-state assignment procedure allowed us to treat ferrous and ferric Fe atoms separately without relying on bond-valence sums. Separating iron-oxidation states independently of bond valence from the outset was critical in revealing two distinct populations within iron(II) and iron(III) sites, a result described in detail below. In short, a significant number of designated iron(II) sites exhibited elevated bond-valence sums around *V_i_ =* 3.5 when a single *R*_0_ parameter was used, which is seen as a minor but clear peak in the bond-valence sum distribution (Fig. 1 ▸ *a*). Such a clear split prompted us to assume two populations with different *R*_0_ parameters for iron(II)–nitrogen bonds. For other iron(II) bonds, a single parameter was sufficient to explain their BVS distributions. The same effect also seems to be present for iron(III)–nitrogen bonds, and we were able to similarly discriminate two populations by splitting the iron(III)–nitrogen *R*_0_ parameter.

### 2.4. Optimizing iron *R*_0_ values using both homoleptic and heteroleptic sites   

Two data sets, corresponding to iron(II) and iron(III), were used as input to separately optimize the corresponding *R*_0_ parameters, with initial values adopted from the literature (Brese & O’Keeffe, 1991 ▸). For a single heteroleptic site, the set of *R*_0_ values that satisfies the bond-valence sum rule is not unique. However, for a group of heteroleptic sites, a set of *R*_0_ values and its corresponding uncertainty can be optimized. Assuming an iron of type α [iron(II), iron(III) or any other variation] and a ligand of type β (presently N, O, F, S, Cl or Br), one may treat *V_i_* (2) as a function of the parameters for all bond types {*R*_0_^αβ^}, with the set of distances between iron and ligand atoms {*d_ij_*} and *b* being fixed in a system of equations. For each iron of type α (differing by oxidation state, spin state or something else), the summed squared deviation around the expected oxidation state, σ^2^ = 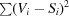, can be minimized against any number of homoleptic and heteroleptic sites. In this way, an optimal set of parameters {*R*_0_^αβ^} for a particular iron type α will be determined for the selection of structures used.

Since σ^2^ is a smooth and convex function of the optimized parameters and we are looking for a minimum that is most likely to be close to the starting point (the reference values previously established in the literature are reasonable), it is convenient to use the nonlinear conjugate-gradient method to converge to a solution. Our numerical procedure is conceptually similar to the approach adopted in a previous study (Liu & Thorp, 1993 ▸), where a damped least-squares method was used to fit three *R*_0_ parameters simultaneously to a number of similar binding sites. In our case, the data set for each iron type is much more varied, with binding sites that contain different numbers and types of ligands. We examine the properties of the optimized parameters in more detail when discussing the results for various iron types.

Our strategy is novel in another way, in fact unprecedented in the literature and contrary to the traditional approach for deriving bond-valence parameters. Namely, we do not derive parameters from a curated set of trusted binding sites. Rather, we adopt the data-driven paradigm and start with all available binding sites of a particular type, apply constraints to filter out the most unreliable sites (a procedure that can be improved in the future) and perform a preset optimization scheme. While we manually investigated many binding sites in the course of this study in order to test and monitor the workflow or to evaluate our results and outliers, in general the sheer number of sites included in the analysis makes it unfeasible to examine all sites individually. This incurs some dangers, especially to the uncertainty of our results, but it also provides some unique advantages. It makes our approach amenable to incremental adjustments, for example by adding additional filters for unreliable sites or by applying updated oxidation-state assignments. By deriving parameters in an automated way our method is readily reproducible, and consequently is easier to extend and improve in the future. For example, it would also be quite straightforward to extend the optimization procedure to include new input structures or additional atom types, to refine the *b* parameter, or even to explore alternative mathematical expressions of the bond length–valence correlation.

One problem is the potential for systematic errors with non-normal or asymmetric distributions. Such bias could result from a number of chemical effects specific to a particular iron type or ligand type. For example, the delocalization of valence electrons will always decrease the bond-valence sum. The only way to deal with such errors is to isolate cases where they appear and discard them during the filtering phase (validation step). For this and similar reasons, instead of standard deviations, we estimate an upper bound on the uncertainty related to each binding site by calculating the change Δ*R*_0_ needed in any one parameter in order to make the calculated bond-valence sum equal to the expected oxidation state,
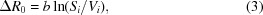
and this value will never be smaller than the standard deviation of *R*_0_, assuming that there are no significant numerical compensation effects. Accordingly, the average of |Δ*R*_0_|^2^ provides a reasonable upper bound on the variance for *R*_0_ and will depend only on the distribution of bond-valence sums. We use |Δ*R*_0_|^2^ as the uncertainty in Tables 1 ▸ and 2 ▸.

### 2.5. Deriving *R*_0_ bond-valence parameters for sodium(I), magnesium(II), potassium(I), calcium(II) and zinc(II)   

The bond-valence *R*_0_ parameters for several cations relevant to macromolecules, namely Na, Mg, K, Ca and Zn, were derived in the same way as described for iron as a control to test our numerical procedure. No cations from this set exhibit multiple oxidation states and therefore they do not need to be divided into subsets. Again, all binding sites that passed the filtering steps (i)–(iii) (§[Sec sec2.2]2.2) were used for *R*_0_ parameter optimization assuming the usual oxidation state. We derived the bond-valence *R*_0_ values for these metals and, as expected, the values correspond to those reported in the literature, within ±0.1 Å of the uncertainty estimated by |Δ*R*_0_|^2^.

## 3. Results and discussion   

### 3.1. First coordination sphere bond-valence *R*_0_ parameters for iron(II) and iron(III)   

The derivation of *R*_0_ parameters in the past has for the most part been based on homoleptic metal-binding sites (Shields *et al.*, 2000 ▸). The few reports including heteroleptic sites do employ optimization techniques, nonetheless they are based on a small selection of manually chosen structures (Liu & Thorp, 1993 ▸). The method we adopted allows the simultaneous refinement of multiple *R*_0_ parameters based on a large set of varied structures, including heteroleptic binding sites. In all cases, the increase in sample size compared with the homoleptic subset is significant, and often a larger sample size is crucial to obtain statistical significance (Table 1 ▸). The significant increase in sample size is also important for some specific types of iron–ligand interactions that occur rarely in the experimental structures deposited in the CSD (such as Fe^II^—F and Fe^III^—F). In such cases, there are insufficient data for a proper *R*_0_ estimation using only homoleptic sites.

It is intuitive that using the same *R*_0_ parameters to model both iron(II) and iron(III) bonds would result in a bimodal distribution of calculated bond-valence sums (*V_i_*), with a clear separation between peaks corresponding to different oxidation states. Clearly, the observation of a bimodal distribution in any other context would also suggest that we are dealing with two subpopulations that should be described by different parameters. The oxidation-state assignment process outlined in §[Sec sec2.3]2.3 allows us to divide our input structures into ferrous and ferric subsets upfront and model the two populations independently. This initial assignment results in a major peak around the expected oxidation state for both iron types. Nonetheless, significant peaks for larger BVS values remain, and this is particularly pronounced in the case of ferrous iron (Fig. 1 ▸ *a*).

We looked into an example structure that contains iron-binding sites from both BVS peaks [CSD Refcode ABEQUV, a di-iron(II) triazole complex], and noticed that there are two different iron(II) sites (Kitchen *et al.*, 2011 ▸). Although both N-homoleptic iron(II) sites (labeled Fe1 and Fe2) are assigned an oxidation state of 2 and each one has six N atoms in the first coordination sphere, one of them (Fe1) has all Fe^II^—N distances in the range 1.911–2.067 Å, while the second (Fe2) has all Fe^II^—N distances in the range 2.146–2.299 Å (Fig. 1 ▸ *b*). The chloride ions near the second iron site probably affect its spin-crossover properties (Kitchen *et al.*, 2011 ▸) and the two iron(II) centers exhibit a spin-crossover transition.

This observation prompted us to raise the hypothesis that the iron spin state would affect the Fe^II^—N bond distance and thus impact the derived *R*_0_ parameters. A manual inspection of the sites from the higher BVS peak of the Fe^II^—N distribution reveals that they were reported as low-spin-state iron. To systematically confirm this hypothesis, a set of iron(II)-binding sites whose spin state is reported in the literature was selected by screening for the presence of the keywords ‘spin state’ and ‘spin crossover’ in the abstract, followed by manual examination of the literature to confirm the spin states of iron(II) sites.

**Figure 2:**
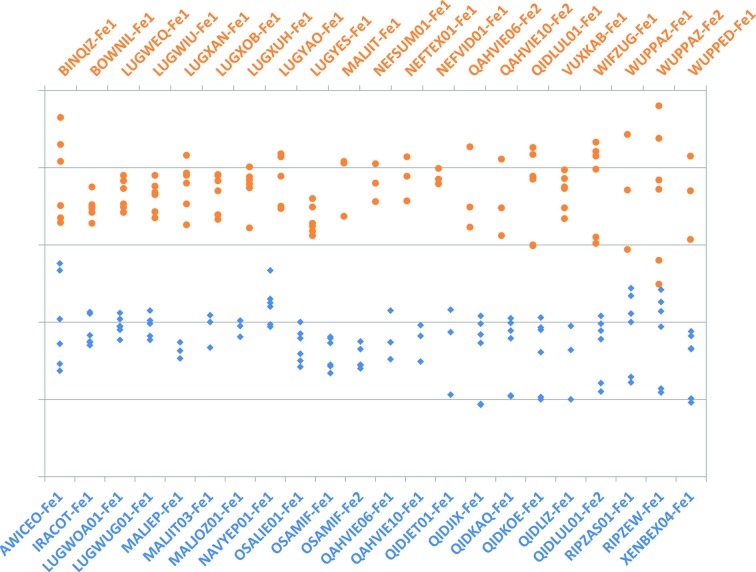
Distribution of distances from iron(II) to nitrogen in six-coordinated iron(II) sites with the iron reported to be either in a low-spin state (shown in blue) or in a high-spin state (shown in orange). For each iron(II) site identified by CSD Refcode and iron(II) atom label, the distances to all six nitrogen ligands are shown. Typical Fe^II^—N distances range between 1.9 and 2.1 Å for sites with a reported low-spin iron, while typical Fe^II^—N distances range between 2.1 and 2.3 Å for sites with a reported high-spin iron.

Further examination of this set of sites confirms that there is a precise correlation between iron(II) spin state and Fe^II^—N bond distance and therefore appropriate *R*_0_ parameters. All of the iron(II)-binding sites from the minor peak of the bimodal BVS distribution whose spin states we could find in the literature were reported to be in a low-spin state and exhibited Fe^II^—N distances in the range 1.9–2.1 Å. On the other hand, all iron(II)-binding sites selected from the major peak that were reported as high spin have larger Fe^II^—N distances in the range 2.1–2.3 Å (Fig. 2 ▸). This correlation clearly justifies resolving two *R*_0_ parameters for Fe^II^—N bonds owing to the presence of two species of iron(II) in different spin states. As a result, Fe^II^—N bonds from low-spin iron sites were refined to an *R*_0_ of 1.57 Å, while Fe^II^—N bonds from high-spin iron sites were refined to an *R*_0_ of 1.76 Å (Table 1 ▸).

A minor peak can also be observed in the BVS distribution for Fe^III^—N when one *R*_0_ parameter is used, and therefore we employed a similar strategy in this case, fitting two independent *R*_0_ parameters. Again, Fe^III^—N bonds best described by an *R*_0_ of 1.70 Å are found to be correlated with the low-spin state (LS), while sites better described by an *R*_0_ of 1.83 Å are predominantly in a high-spin state (HS). Another nomenclature to annotate spin state uses singlet, triplet and quintet for the iron(II) ion or doublet, quartet and sextet for the iron(III) ion. The correspondence between the HS/LS states and these spin states for various iron-containing compounds can be found in the literature (Porro *et al.*, 2009 ▸; Swart, 2008 ▸).

In fact, using a different approach as part of her PhD study, Dr Elna Pidcock from the University of Manchester had also observed two distinct populations of Fe—N distances that can be described precisely by different iron spin states for both iron(II) and iron(III) (Pidcock, 1995 ▸). This independent research provides further evidence about the reproducibility of the results from our analytical and data-mining procedures.

Table 1 ▸ summarizes the optimized *R*_0_ parameters for iron(II) and iron(III), estimated using both homoleptic and heteroleptic metal-binding sites within the first coordination sphere, and compares them with several previous studies (Brese & O’Keeffe, 1991 ▸; Liu & Thorp, 1993 ▸; Kanowitz & Palenik, 1998 ▸). While most of the *R*_0_ parameters derived here for iron agree within ±0.1 Å with previously reported values, we found that the Fe—N *R*_0_ parameters reported previously appear to agree well only with the corresponding high-spin (HS) species. The newly determined *R*_0_ values for low-spin (LS) species exhibit a significant separation from their counterparts in high-spin (HS) species, namely 0.19 Å for Fe^II^—N and 0.13 Å for Fe^III^—N.

The different bond-valence *R*_0_ values for iron in different spin states are in line with the different ionic radii associated with these spin states. For six-coordinated iron(II), the effective ionic radii was reported to be 0.78 and 0.61 Å for high and low spin, respectively, with a separation of 0.17 Å (Shannon, 1976 ▸). For six-coordinated iron(III), the effective ionic radii was reported to be 0.65 Å for high spin and 0.55 Å for low spin, with a separation of 0.10 Å. The difference in effective ionic radii is likely to reflect the same physical effect as the difference in *R*_0_ parameters that we obtained for different spin states. We were able to distinguish high-spin (HS) and low-spin (LS) bond valences only for Fe—N bonds, but not for iron with other ligands, possibly owing to the small number of available structures in an alternate spin state.

**Figure 3:**
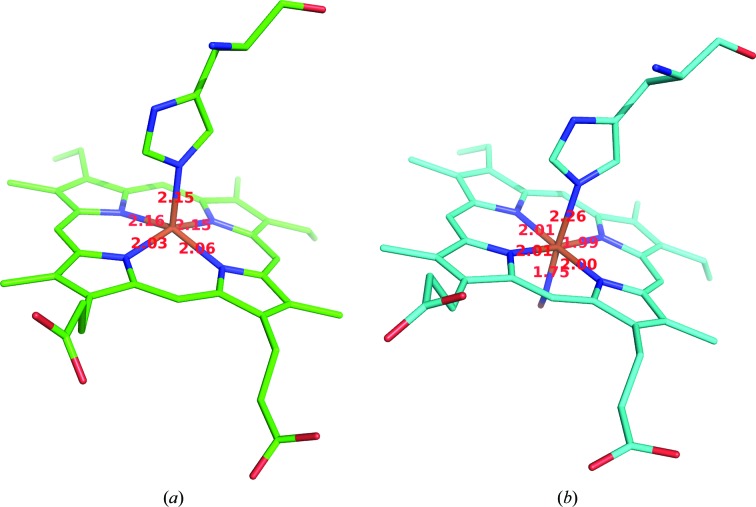
Examples of Fe—N sites in the PDB, with Fe atoms shown in brown, O atoms shown in red and N atoms shown in blue. C atoms are shown in different colors for the different iron-binding sites. (*a*) Heme with high-spin iron(II), C atoms shown in green; (*b*) heme with low-spin iron(III), C atoms shown in cyan.

The significantly different *R*_0_ parameters for Fe^III^—N derived here have some practical biological implications. For comparison, it is worth noting that these differences are larger than the theoretical difference in *R*_0_ to mistakenly assign iron(III) to oxidation state 4 as iron(IV): Δ*R*_0_(III→IV) = *b* × ln(4/3) = 0.106 Å < 0.16 Å. This suggests that the previously reported values may lead to an incorrect estimation of the oxidation state in iron-containing proteins, including hemoglobin. Iron has been established to be in the low-spin ferric form [LS iron(III)] in the oxidized form of hemoglobin (oxyhemoglobin) by X-ray photoelectron spectroscopy and X-ray absorption near-edge structure (XANES) studies at the Fe *K* edge (Pin *et al.*, 1982 ▸; Bianconi *et al.*, 1985 ▸). However, applying the literature reference *R*_0_ parameter for iron(III) (1.815–1.86 Å; Liu & Thorp, 1993 ▸; Brese & O’Keeffe, 1991 ▸) to the metal geometry observed in protein crystal structures of oxyhemoglobin results in a BVS of 3.90–4.41 (Table 3 ▸). In contrast, using the *R*_0_ parameter in the low-spin state for Fe^III^—N derived here (1.70 Å) leads to a BVS of 2.86 [*i.e.* iron(III)], which is much closer to the cited experimental value (Chan *et al.*, 1998 ▸). A similar correction can also be applied to the iron-binding site in deoxyhemoglobin, which tends to exist in a high-spin configuration (Fermi *et al.*, 1984 ▸). Namely, the previously reported *R*_0_ parameter for Fe^II^—N bonds (1.86 Å; Brese & O’Keeffe, 1991 ▸) results in a BVS of 2.58, while using our *R*_0_ parameter in the high-spin state yields a BVS of 1.98. Although many cases can be found where these new values could be of use, for us improvements in the interpretation of protein iron-binding sites are the most convincing arguments for adopting these revised values for Fe—N bond-valence *R*_0_ parameters (Fig. 3 ▸).

### 3.2. Validation of the procedure by the derivation of *R*_0_ parameters for Na, Mg, K, Ca and Zn as control data sets   

The validity of our procedure is grounded on its ability to reproduce the well recognized and widely used set of reference bond-valence *R*_0_ parameters (Brese & O’Keeffe, 1991 ▸). The data set used for our validation procedure is also generated by the incorporation of heteroleptic sites that significantly increased the sample size for our validation set of biologically relevant metal ions. For example, the sample size for zinc more than tripled when adding heteroleptic sites. For the selected metals (Na, Mg, K, Ca and Zn) and ligands (N, O, F, S, Cl and Br), eight pairs of metal–ligand interactions occur so rarely in the experimentally observed metal-binding sites in CSD that there are insufficient data for proper *R*_0_ estimation from homoleptic sites. In most cases the situation is improved after adding heteroleptic data, although some sample sizes were still unreliably small (in particular Ca—F).

The final *R*_0_ parameters determined by our optimization procedure (Table 2 ▸) agree with the reference values within either the estimated upper bound of the uncertainty or 0.1 Å (which we considered as a rough limit for significance). There are discrepancies with the reference values owing to inadequacies in our input data validation and small sample sizes, but these factors are usually manifest themselves in the variation of our uncertainty estimates. It has also been noted that differences in derived bond-valence parameters between the ICSD and CSD [*i.e.* *R*_0_(inorg) − *R*_0_(org)] range from −0.02 to +0.05 Å (Brown, 2009 ▸). Differences in the data sets and protocols used for deriving *R*_0_ are probably insignificant in our case and most discrepancies fall within the estimated upper bound of standard deviation. Since there is still room for improvement of the data-preprocessing procedure, more rigorous filtering of the input structures to eliminate binding sites that are not suitable for parameterizing the bond-valence model could further reduce the uncertainties and discrepancies.

In fact, we are encouraged by how closely our optimized values for *R*_0_ follow the reference parameters, despite largely disjoint samples (organic *versus* inorganic ligands), fundamentally different protocols (including heteroleptic sites) and distinct selection strategies (automated filtering *versus* manual curation). The quality of the correspondence with reference values also demonstrates that the bond-valence model is surprisingly generic and the *R*_0_ parameters determined based on a limited set of carefully chosen chemical structures two decades ago (Brese & O’Keeffe, 1991 ▸; Brown & Altermatt, 1985 ▸) are still highly accurate (on average). Nonetheless, we believe that with improvements to the validation phase and optimization procedure, and perhaps the inclusion of additional structures, a data-driven approach can help to discover more results that are chemically meaningful such as the spin-state differentiation described here.

### 3.3. Application of *R*_0_ parameters in metal-binding-site modeling in macromolecular crystallography   

Using the *R*_0_ parameters derived here, it is straightforward to derive a set of ideal metal-ligand distances for a binding site with a number of equal bond-valence contributions. For example, in sites with octahedral geometry, each ligand should contribute a bond valence of 1/6 to monovalent metals (Na, K) or 2/6 to divalent metals (Mg, Ca). According to (2), the corresponding typical metal–ligand distances are summarized in Table 4 ▸, as derived from the *R*_0_ values in Table 2 ▸, and agree well with metal–ligand distances reported previously (Bergerhoff & Brandenburg, 2004 ▸).

Iron–ligand distances show considerable variation, and iron-binding sites are commonly observed in many different configurations that could impact the iron–ligand distance. These configurations are affected by various different factors, including different coordination number (five or six), oxidation state (II or III) and spin state (low spin or high spin), and consequently there is no single typical distance (Table 5 ▸). As we have demonstrated earlier, iron in heme may display different iron–nitrogen distances depending not only on the oxidation state of the central iron but also on the spin state of the central iron. Moreover, numerous complexes containing iron–sulfur interactions often exhibit a tetrahedral geometry instead of an octahedral geometry, resulting in an Fe—S distance of around 2.27–2.36 Å (Table 5 ▸). We have also noticed that the bond-valence *R*_0_ parameters derived for tetrahedral Fe^III^—S and Fe^II^—S gave inaccurate results for oxidation-state calculations in many cases over the whole range of coordination numbers other than 4 (coordination number = 2, 3, 5, 6). The lack of a commonly applicable bond-valence parameter has been reported previously (Pidcock, 1995 ▸).

While these typical distances can be used to substitute or update individual metal–ligand restraint distances for refining models of metal-binding sites in macromolecule refinement programs such as *REFMAC* (Murshudov *et al.*, 2011 ▸), it would be appropriate to introduce additional restraints based on the overall bond-valence sum for each metal-binding site during crystallographic refinement. In particular, in cases where individual metal–ligand distances alone are different, restraints based on bond-valence sums could be used to guess the identity of metal-binding sites in macromolecular structures.

Using the updated set of bond-valence parameters derived here, we have recently launched a web-based service to provide metal-binding site validation in macromolecular structures (Zheng *et al.*, 2014 ▸). The bond-valence sum method has previously been shown to be applicable to metal-binding sites present in macromolecular crystal structures (Müller *et al.*, 2003 ▸). With the data reported here showing that both the bond-valence model and the set of previously derived bond-valence *R*_0_ parameters (Brese & O’Keeffe, 1991 ▸) align well with newly derived *R*_0_ parameters from a diverse set of crystal structures containing metal–organic sites (Allen, 2002 ▸), we are more confident applying these values for metal-ion binding sites in macromolecular crystallography.

## 4. Conclusion   

By minimizing the squared deviations of bond-valence sums around the expected oxidation state, we derived optimal *R*_0_ bond-valence parameters from a large set of iron–organic binding sites from the CSD. Several thousand homoleptic and heteroleptic iron-binding sites were treated together during each optimization. We were able to discern two populations of iron(II)-binding sites, corresponding to iron–nitrogen *R*_0_ parameters of 1.57 Å for low-spin iron and 1.76 Å for high-spin iron. Iron(III) sites revealed a similar bimodal distribution, corresponding to iron–nitrogen *R*_0_ parameters of 1.70 and 1.83 Å for low-spin and high-spin iron, respectively. To validate our novel approach, we examined its applicability to five other biologically relevant metal ions (Na, Mg, K, Ca and Zn). All of the resulting metal–ligand bond-valence parameters and distances agree with the *R*_0_ values reported previously within the estimated uncertainty |Δ*R*_0_|^2^ of 0.1 Å. We recommend the use of spin-state-dependent *R*_0_ values for evaluating future structures with metal–organic sites containing iron–nitrogen bonds, particularly in heme-containing proteins. Our data-driven optimization procedure is fully reproducible and therefore provides a starting point for further improving the methodology and further refinement of bond-valence model parameters. The data and code for the numeric procedure for optimization of bond-valence parameters has been made available on github at https://github.com/MinorLabUVa/bvparm-metalorganics and on figshare at https://dx.doi.org/10.6084/m9.figshare.964285.v3.

## Figures and Tables

**Table 1:** Optimized *R*_0_ parameters (in Å) derived for iron(II) and iron(III), compared with reference literature values (Brese & O’Keeffe, 1991 ▸; Liu & Thorp, 1993 ▸; Kanowitz & Palenik, 1998 ▸) For each metal–ligand pair, *R*_0_ is the mean of parameters calculated separately from all validated homoleptic sites (with the standard deviation in parentheses) and *R*_0_ (CSD) denotes the value obtained from our optimization procedure (with the average of |Δ*R*_0_|^2^ as an upper bound on the uncertainty in parentheses) calculated over all validated homoleptic and heteroleptic sites. CN is the coordination number of the first coordination sphere.

Ligands to iron	N	O	F	S (CN = 4)	Cl	Br
Iron(II)						
No. of homoleptic sites	497; 751	378	0	159	68	11
*R*_0_ (Å)	1.57 (2); 1.76 (2)	1.71 (4)	—	2.08 (9)	2.04 (4)	2.20 (1)
Total No. of sites	—	1144	34	—	518	65
*R*_0_ (CSD) (Å)	—	1.70 (4)	1.67 (4)	—	2.05 (3)	2.21 (2)
Brese	1.86	1.734	1.65	2.16	2.06	2.26
Liu	1.769	1.700	—	2.125	—	—
Kanowiz		1.713	—	—	—	—
Iron(III)						
No. of homoleptic sites	114; 62	665	14	68	280	59
*R*_0_ (Å)	1.70 (2); 1.83 (3)	1.75 (4)	1.68 (1)	2.10 (11)	2.08 (2)	2.22 (1)
Total No. of sites	—	2663	60	—	1034	104
*R*_0_ (CSD) (Å)	—	1.76 (3)	1.67 (4)	—	2.09 (2)	2.23 (4)
Brese	1.86	1.759	1.67	2.16	2.09	2.26
Liu	1.815	1.765	—	2.134	—	—
Kanowitz	—	1.751	—	—	—	—

**Table 2:** Optimized *R*_0_ parameters (in Å) for bonds to Na, Mg, K, Ca and Zn using first coordination sphere ligand atoms For each metal–ligand pair, three values are reported: *R*_0_ is the mean of parameters calculated separately from all validated homoleptic sites (with the standard deviation in parentheses), *R*_0_ (CSD) denotes the value obtained from our optimization procedure (with the average of |Δ*R*_0_|^2^ as the upper bound on the uncertainty in parentheses) calculated over all validated homoleptic and heteroleptic sites, and *R*_0_ (Brese) is the value previously reported in the literature (Brese & O’Keeffe, 1991 ▸).

Ligands	N	O	F	S	Cl	Br
Na						
No. of homoleptic sites	60	1480	2	1	2	0
*R*_0_ (Å)	1.91 (9)	1.77 (9)	1.69 (2)	2.27	2.16 (1)	—
Total No. of sites	702	2404	75	126	87	15
*R*_0_ (CSD) (Å)	1.88 (8)	1.75 (9)	1.67 (5)	2.23 (8)	2.16 (4)	2.32 (6)
*R*_0_ (Brese) (Å)	1.93	1.80	1.677	2.28	2.15	2.33
Mg						
No. of homoleptic sites	137	613	0	0	4	3
*R*_0_ (Å)	1.81 (6)	1.67 (4)	—	—	2.07 (1)	2.23 (2)
Total No. of sites	509	1075	9	28	90	66
*R*_0_ (CSD) (Å)	1.78 (6)	1.67 (4)	1.64 (1)	2.20 (4)	2.11 (3)	2.26 (2)
*R*_0_ (Brese) (Å)	1.85	1.69	1.58	2.18	2.08	2.28
K						
No. of homoleptic sites	69	876	3	9	7	1
*R*_0_ (Å)	2.31 (15)	2.10 (8)	2.18 (6)	2.74 (11)	2.48 (6)	2.69
Total No. of sites	950	2123	101	170	107	17
*R*_0_ (CSD) (Å)	2.22 (8)	2.07 (7)	2.03 (5)	2.62 (7)	2.49 (4)	2.63 (5)
*R* (Brese) (Å)	2.26	2.13	1.99	2.59	2.52	2.66
Ca						
No. of homoleptic sites	53	475	0	0	0	0
*R*_0_ (Å)	2.08 (6)	1.94 (5)	—	—	—	—
Total No. of sites	316	801	2	15	31	14
*R*_0_ (CSD) (Å)	2.07 (5)	1.93 (4)	1.89 (1)	2.42 (4)	2.33 (1)	2.51 (2)
*R*_0_ (Brese) (Å)	2.14	1.96	1.84	2.45	2.37	2.49
Zn						
No. of homoleptic sites	1393	2328	0	251	439	50
*R*_0_ (Å)	1.75 (3)	1.69 (3)	—	2.09 (2)	2.01 (1)	2.15 (2)
Total No. of sites	9275	8811	18	1330	20549	403
*R*_0_ (CSD) (Å)	1.75 (3)	1.69 (2)	1.64 (2)	2.09 (2)	2.01 (1)	2.14 (1)
*R*_0_ (Brese) (Å)	1.77	1.74	1.62	2.09	2.01	2.15

**Table:** (*a*) PDB entry 2hhb (1.74 Å), deoxyhemoglobin, HS iron(II).

Ligands	N1	N2	N3	N4	N5	O6	BVS
Distance (Å)	2.03	2.06	2.15	2.15	2.16	3.39	
v.u.(*R*_0_ = 1.57 Å) (LS)	0.29	0.27	0.21	0.21	0.20	0.01	1.19
v.u.(*R*_0_ = 1.76 Å) (HS)	0.48	0.44	0.35	0.35	0.34	0.01	1.98
v.u.(*R*_0_ = 1.86 Å) (Brese)	0.63	0.58	0.46	0.46	0.44	0.02	2.59

**Table:** (*b*) PDB entry 1buw (1.90 Å), oxyhemoglobin, LS iron(III).

Ligands	N1	N2	N3	N4	N5	N6	BVS
Distance (Å)	1.75	1.99	2.00	2.01	2.01	2.26	
v.u.(*R*_0_ = 1.70 Å) (LS)	0.87	0.46	0.44	0.43	0.43	0.22	2.86
v.u.(*R*_0_ = 1.83 Å) (HS)	1.24	0.65	0.63	0.61	0.61	0.31	4.06
v.u.(*R*_0_ = 1.86 Å) (Brese)	1.35	0.70	0.68	0.67	0.67	0.34	4.41
v.u.(*R*_0_ = 1.815 Å) (Liu)	1.19	0.62	0.61	0.59	0.59	0.30	3.90

**Table 4:** Typical first coordination sphere distances for Na, Mg, K, Ca and Zn derived from the converged *R*_0_ values in Table 3 ▸ CN is the coordination number of the first coordination sphere. Octahedral geometry for Na, Mg, K and Ca and tetrahedral geometry for Zn are assumed, with equal bond-valence contributions from each atom. Distances are calculated using the *R*_0_ values derived in this study (when sufficient validated data from the CSD were available).

Distance (Å)	N	O	F	S	Cl	Br
Na (CN = 6)	2.54	2.41	2.33	2.89	2.82	2.98
Mg (CN = 6)	2.19	2.08	2.05	2.61	2.52	2.67
K (CN = 6)	2.88	2.73	2.69	3.28	3.15	3.29
Ca (CN = 6)	2.48	2.34	2.30	2.83	2.74	2.92
Zn (CN = 4)	2.01	1.95	1.90	2.35	2.27	2.40

**Table 5:** Typical first coordination sphere distances for Fe derived from the converged *R*_0_ values in Table 1 ▸ CN is the coordination number of the first coordination sphere. CN = 4 is assumed for Fe—S sites and CN = 6 is assumed for iron sites interacting with non-sulfur ligands, with equal bond-valence contributions from each atom. Distances are calculated using the *R*_0_ values derived in this study (when sufficient validated data from the CSD were available).

Distance (Å)	N (CN = 6)	O (CN = 6)	F (CN = 6)	S (CN = 4)	Cl (CN = 6)	Br (CN = 6)
LS iron(II)	1.98	2.11	2.08	2.33	2.46	2.62
HS iron(II)	2.17					
LS iron(III)	2.11	2.17	2.08	2.35	2.50	2.64
HS iron(III)	2.24					
